# VEBA: a modular end-to-end suite for in silico recovery, clustering, and analysis of prokaryotic, microeukaryotic, and viral genomes from metagenomes

**DOI:** 10.1186/s12859-022-04973-8

**Published:** 2022-10-12

**Authors:** Josh L. Espinoza, Chris L. Dupont

**Affiliations:** 1 Department of Environment and Sustainability, J. Craig Venter Institute, 4120 Capricorn Ln, La Jolla, CA 92037 USA; 2 Department of Human Biology and Genomic Medicine, J. Craig Venter Institute, La Jolla, CA 92037 USA

**Keywords:** Metagenomics, Pipeline, Binning, Metagenome-assembled genome

## Abstract

**Background:**

With the advent of metagenomics, the importance of microorganisms and how their interactions are relevant to ecosystem resilience, sustainability, and human health has become evident. Cataloging and preserving biodiversity is paramount not only for the Earth’s natural systems but also for discovering solutions to challenges that we face as a growing civilization. Metagenomics pertains to the in silico study of all microorganisms within an ecological community in situ*,* however, many software suites recover only prokaryotes and have limited to no support for viruses and eukaryotes.

**Results:**

In this study, we introduce the *Viral Eukaryotic Bacterial Archaeal* (VEBA) open-source software suite developed to recover genomes from all domains. To our knowledge, *VEBA* is the first end-to-end metagenomics suite that can directly recover, quality assess, and classify prokaryotic, eukaryotic, and viral genomes from metagenomes. *VEBA* implements a novel iterative binning procedure and hybrid sample-specific/multi-sample framework that yields more genomes than any existing methodology alone. *VEBA* includes a consensus microeukaryotic database containing proteins from existing databases to optimize microeukaryotic gene modeling and taxonomic classification. *VEBA* also provides a unique clustering-based dereplication strategy allowing for sample-specific genomes and genes to be directly compared across non-overlapping biological samples. Finally, *VEBA* is the only pipeline that automates the detection of candidate phyla radiation bacteria and implements the appropriate genome quality assessments. *VEBA*’s capabilities are demonstrated by reanalyzing 3 existing public datasets which recovered a total of 948 MAGs (458 prokaryotic, 8 eukaryotic, and 482 viral) including several uncharacterized organisms and organisms with no public genome representatives.

**Conclusions:**

The *VEBA* software suite allows for the in silico recovery of microorganisms from all domains of life by integrating cutting edge algorithms in novel ways. *VEBA* fully integrates both end-to-end and task-specific metagenomic analysis in a modular architecture that minimizes dependencies and maximizes productivity. The contributions of *VEBA* to the metagenomics community includes seamless end-to-end metagenomics analysis but also provides users with the flexibility to perform specific analytical tasks. *VEBA* allows for the automation of several metagenomics steps and shows that new information can be recovered from existing datasets.

**Supplementary Information:**

The online version contains supplementary material available at 10.1186/s12859-022-04973-8.

## Introduction

The importance of microorganisms and how their interactions are relevant to ecosystem resilience, sustainability, and human health has become more apparent with each study conducted. Therefore, cataloging and preserving biodiversity is paramount not only for the Earth’s natural systems but also for discovering solutions to challenges that we face as a growing civilization in the midst of global pandemics and a warming climate. Large scale microbiome surveys have been enacted for cataloging and describing the human microbiome (Human Microbiome Project (HMP) [1, 2]), environmental taxonomic profiling (Earth Microbiome Project (EMP) [3]), the world’s oceans (Tara [4], GOS [5]), and, perhaps the most ambitious, the sequencing of all eukaryotes (Earth BioGenome Project (EBP) [6]).

Microorganisms provide humanity with potential solutions to some of our most complex geopolitical and socioeconomic challenges. For instance, all domains of microorganisms have been harnessed for progressing medicine including antimicrobial compounds derived from prokaryotes [7–10], bacteriophage therapy developed from viruses [11], and yeast that could engineer drugs with complex glycans [12]. In addition to biomedical applications, microorganisms have been reengineered for biofuel production [13–15], beverage fermentation [16], waste water treatment [17], sustainable agriculture [18], and self-repairing building materials [19, 20].

Metagenomics is a sequencing-based microbial-centric survey of an ecosystem often composed of prokaryotes, eukaryotes, and viruses. There are 3 main approaches to metagenomics each with their own strengths/weaknesses, resource demand, and capacity for investigating different hypotheses. The first approach to metagenomics is the marker-gene survey where predefined primers are used to amplify specific fragments of genetic material from an environmental sample. These primers typically amplify ribosomal DNA (e.g., 16S in prokaryotes or 18S in eukaryotes) to produce either amplicon sequence variants [21] or clusters of operational taxonomic units [22] that are interpreted as taxonomic barcodes classified based on a reference database. While marker-gene survey classification is reference dependent, novelty can be flagged post hoc if a query sequence is divergent enough from other sequences in the reference. The biggest caveat of marker-gene surveys is that they provide no phylogenetic resolution nor insight into function, although, well characterized environments such as the human gut can benefit from functional inference software [23]. The second approach is read-based shotgun metagenomics which involves a reference database, aligning fastq reads to said reference, and generating counts tables with respect to taxonomic features in the reference [24–26]. Read-based approaches have phylogenetic resolution but is decoupled from function. The benefits of read-based approaches are that the algorithms are easy to implement, scalable to large datasets, and have rapid run times but are entirely dependent on a reference and cannot be used de novo*.* The third approach is assembly-centric shotgun metagenomics where, in short, reads are assembled into contigs, metagenome-assembled genomes (MAG) are binned from assemblies, genes are modeled, and annotation/classification is performed. Assembly-centric metagenomics is far more computationally challenging but provides vastly more power in terms of biological interpretation having led to the characterization of uncultivated lineages vastly expanding the tree of life and finding potential links in eukaryogenesis [27, 28]. In particular, assembly-centric metagenomics allows for coupling taxonomy with function and is not dependent on—though, supplemented by—existing reference databases as is required for read-based metagenomics. However, the majority of software packages and suites for recovering genomes from metagenomes perform exclusively on prokaryotes [29, 30]. Recently, the advent of robust viral genome recovery software has broken barriers in viral metagenomics [31, 32] but these standalone packages are not implemented in many metagenomic pipelines and, thus, need to be run independently. As far as we know, there exists no published software suite that recovers eukaryotic genomes from metagenomes, models eukaryotic genes with intron structure, and classifies taxonomy.

Microeukaryotes are largely ignored from assembly-centric studies for a variety of reasons including binning algorithms being developed exclusively for prokaryotes [33, 34], gene modeling software with inconvenient licensing agreements making installation a significant barrier for entry [35], or software that requires lineage-specific references making automation difficult for de novo metagenomics [36]. Recent studies have demonstrated the merit of recovering microeukaryotes from metagenomes [37, 38]; while essential to the field, these methods are currently are not autonomous and require expert curation during the analysis and assessment phases making reproducibility and large-scale implementation on new or existing datasets difficult.

Recently, there has been an explosion in software developed to handle prokaryotic genomes with a multitude of binning algorithms [33, 39, 40], consensus binning methodologies to utilize the strengths of each binning algorithm [29, 34], lineage-specific genome quality assessment [41], and consensus genome classification tools [42] making high-quality assembly-centric prokaryotic metagenomics only a *Conda* virtual environment and a few commands away from entry-level computational biologists. Two commonly used metagenomics pipelines, *MetaWRAP* [29] and *SqueezeMeta* [30], perform exclusively on prokaryotic organisms, do not properly account for candidate phyla radiation (CPR), and discard unbinned contigs after a single pass; potentially failing to maximize the information gain from a given dataset. *MetaWRAP* has set a precedent in end-to-end modular metagenomics suites and is agnostic in its support for sample-specific and multi-sample approaches. However, it is not actively maintained and can be difficult to install due to forcing incompatible package dependencies to work together in a single compute environment. *SqueezeMeta* places a strong emphasis in coassembly-based metagenomics, which can be useful when comparing genomic features between samples that can be difficult in sample-specific metagenomics (a caveat we address in this study). However, coassembly results in composite MAGs that have lost sample specific strain level variations. This composite property of coassembly-based metagenomics was initially noted in marine environments [43, 44] and has since been demonstrated in the oral microbiome [45, 46]. In the past, coassembly was necessary due to a paucity of data but with the decrease in sequencing costs, sample-specific assembly and subsequent genome recovery is possible. However, the challenge remains to collapse similar MAGs into representative features (e.g., species) for comparing abundances between samples while retaining sample-specific resolution on relative data.

In this study, we introduce the *Viral Eukaryotic Bacterial Archaeal* (VEBA) open-source software suite developed with all domains of microorganisms as the primary objective (not post hoc adjustments) including prokaryotic, eukaryotic, and viral organisms. To our knowledge, *VEBA* is the first end-to-end metagenomics software suite that can directly recover and analyze eukaryotic and viral genomes in addition to prokaryotic genomes with automated support for CPR. *VEBA* implements a novel iterative binning procedure and an optional hybrid sample-specific/multi-sample framework that recovers more genomes than non-iterative methods. To optimize microeukaryotic gene calling and taxonomic classification, *VEBA* includes a consensus microeukaryotic database containing protists and fungi compiled from several existing databases. *VEBA* also provides a unique clustering-based dereplication strategy allowing for sample-specific genomes and proteins to be directly compared across non-overlapping biological samples. In addition, *VEBA* is the only pipeline that automates the detection of CPR bacteria and implements the appropriate genome quality assessments for said organisms. Lastly, we demonstrated *VEBA*’s capabilities by reanalyzing 3 existing public datasets and identified several previously uncaptured organisms including eukaryotic and viral organisms with no existing genome representatives. The *VEBA* software suite is open-sourced and freely available (https://github.com/jolespin/veba).

## Methods

### Databases

To build a microeukaryotic protein database that could be used in both environmental and clinical settings, we combined the following databases in the following order: (1) *MMETSP* [47], (2) *EukZoo* [48], *EukProt* [49], and *NCBI non-redundant* [50]. However, these are not simply concatenated databases as each one has been filtered to include only microeukaryotes and fungi with prokaryotes and metazoans removed (Additional file 2: Table S2). As these databases are not mutually exclusive, dereplication by sequences and identifiers was necessary. The identifiers for labels have also been modified for seamless usage and parsing with *MetaEuk* [51]. Lastly, only records associated with source organisms that had lineages characterized up to class were considered as this database is used for both eukaryotic gene modeling and annotation. *MMSeqs2* [52] is used to build the processed microeukaryotic reference database which is compatible with *MetaEuk* for exon-aware gene calls and eukaryotic lineage classification.

Also included with the distribution are 5 marker protein sets included in the distribution: (1) *Archaea_76.hmm* [53, 54]; (2) *Bacteria_71.hmm* [53, 54]; (3) *CPR_43.hmm* [41]; (4) *Fungi_593.hmm* [55]*,* and (5) *Protista_83.hmm* [54, 56] that can be used for phylogenetic inference and other marker-based methodologies such as the developmental branch of *DAS Tool* (https://github.com/cmks/DAS_Tool/tree/dev_customSCG). The remaining databases such *NCBI non-redundant* [50]*, KOFAM* [57]*, Pfam* [58]*, GTDB-Tk* [42]*, CheckM* [41]*, CheckV* [31]*,* and an *ETE3* [59] configured NCBI Taxonomy database are installed separately using a database installation script.

### Workflow architecture

The *GenoPype* Python package (https://github.com/jolespin/genopype) was developed to construct *VEBA* and all the modules that comprise the pipeline. *GenoPype* is a lightweight *Python* library for computational pipelines that splits the workflow into individual steps. Each step of the workflow has a designated set of log files (standard out, standard error, and return codes), checkpoint files for continuing an existing run, an executable of all the commands, and file validation for input and output files. The dependency framework for *VEBA* is built using *Conda* (https://conda.io/), primarily using the *Bioconda* channel [60], where each module is coupled with a specific *Conda* environment and all necessary *Conda* environments are configured using the install script in the GitHub repository.

### *VEBA* utility scripts

*VEBA* comes equipped with several utility scripts that are intended for running automatically in the backend of *VEBA* or seamless transition of data to and from various tools. These scripts include essential post-processing methods such as modifying gene models to include useful identifier information in a file-friendly format, binning wrappers, concatenation methods for various file types, fasta utilities, quality filtering methods, partitioning batch jobs, consensus classification/annotation, and so on. These scripts include a wrapper around *Tiara* [61], a program that predicts taxonomic domain for contigs, which aggregates the prediction probabilities for each domain category into logits and uses a softmax transformation to scale the logits into MAG-level prediction probabilities. The consensus ortholog annotation script uses the natural language processing capabilities of *UniFunc* [62] to compile consensus annotations using individual annotations for each protein in an orthogroup. The consensus genome classification script includes the following algorithm given a table of protein lineage classifications and scores: (1) create an array of *N* scaling factors, determined by the leniency parameter, where *N* represents the number of taxonomic levels; (2) iterate through protein annotation table; (3) use the score provided for the annotation (e.g., bitscore, percent identity) and create a running sum for each taxonomic level for *TaxonLevel*_*0*_*:TaxonLevel*_*i*_ where *i* is in the interval [1,*N*] multiplying scores by the scaling factors; and (4) assign taxonomy to the highest scoring group.

The exhaustive list can be found under the script directory: https://github.com/jolespin/veba/src/scripts

### *VEBA* modules

**Fig. 1:**
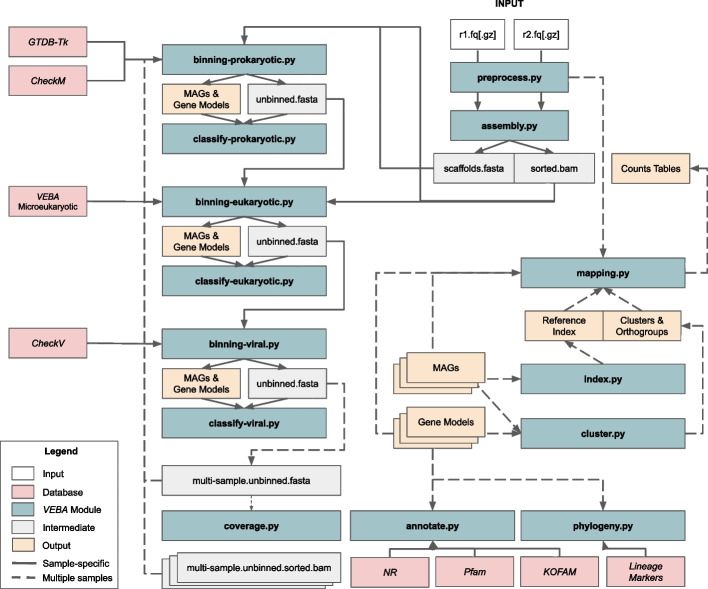
Schematic of *VEBA* workflow. *VEBA* modules and workflow I/O connectivity

*VEBA* is partitioned into several modules each targeting an independent stage of metagenomics. A schematic detailing the flow of information through the pipeline is shown in Fig. 1.

#### *preprocess.py*—Fastq quality trimming, adapter removal, decontamination, and read statistics calculations

The preprocess module is a wrapper around our *fastq_preprocessor* (https://github.com/jolespin/fastq_preprocessor) which is a modernized reimplementation of *KneadData* (https://github.com/biobakery/kneaddata) that relies on *fastp* [63] for ultra-fast automated adapter removal and quality trimming. Pairing of the trimmed reads is assessed and corrected using *BBTools’ repair.sh* (https://sourceforge.net/projects/bbmap). If the user provides a contamination database (e.g., the human reference genome), then trimmed reads are aligned using *Bowtie2* [64] and reads that do not map to the contamination database are stored. If the *--retain_contaminated_reads* flag is used then the contaminated reads are stored as well*.* Similarly, if a *k*-mer reference database is provided (e.g., ribosomal *k-*mers) then the trimmed or decontaminated reads are aligned against the reference database using *BBTools’ bbduk.sh* with an option for storing hits. By default, the none of the contaminated or *k-*mer analyzed reads are stored but regardless of the choice for retaining reads, the read sets are quantified using *SeqKit* [65] for accounting purposes (e.g., % contamination or % ribosomal). All sequences included were downloaded using *Kingfisher (*https://github.com/wwood/kingfisher-download*),* included in the *preprocess* environment*,* which is a fast and flexible program for the procurement of sequencing files and their annotations from public data sources including *ENA, NCBI SRA, Amazon AWS,* and *Google Cloud.*

#### *assembly.py* – Assemble reads, align reads to assembly, and count mapped reads

The assembly module optimizes the output for typical metagenomics workflows. In particular, the module does the following: (1) assembles reads using either *metaSPAdes* [default] [66], *SPAdes* [67], *rnaSPAdes* [68], or any of the other task-specific assemblers installed with the *SPAdes* package [69, 70]; (2) builds a *Bowtie2* index for the *scaffolds.fasta* (or *transcripts.fasta* if *rnaSPAdes* is used); (3) aligns the reads using *Bowtie2* to the assembly; (4) pipes the alignment file into *Samtools* [71] to produce a sorted BAM file (required for coverage applications); (5) counts the reads mapping to each contigs via *featureCounts* [72]; and (6) runs *SeqKit* for useful assembly quality control statistics such as N50, number of contigs, and total assembly size. This module automates many critical yet overlooked workflows dealing with assemblies that are typically performed post hoc such as contig-level sequence alignment, contig-level counts tables, assembly indexing, and assembly statistics.

#### *coverage.py*—Align reads to a (multi-sample/pseudo-coassembly) reference and count mapped reads

The coverage module further optimizes the output for typical metagenomics workflows. In particular, the module does the following: (1) filters contigs based on a size filter (default 1500 bp); (2) builds a *Bowtie2* index for the *reference.fasta*; (3) aligns the reads from all provided samples using *Bowtie2* to the assembly; (4) pipes the alignment file into *Samtools* to produce a sorted BAM file; (5) counts the reads mapping to each contig via *featureCounts*; and (6) *SeqKit* for useful assembly statistics such as N50, number of contigs, and total assembly size [65]. The recommended usage for this module is after prokaryotic, eukaryotic, and viral binning has been performed and the unbinned contigs are merged into a single concatenated reference from multiple samples used as input (i.e., a pseudo-coassembly). The outputs of this module are expected to be used as a final pass through prokaryotic and eukaryotic binning modules successively. While there is overlap in functionality between *coverage.py* and *assembly.py, coverage.py* was designed for multi-sample coverage calculations and does not perform assembly (Fig. 1); although, it supports single sample coverage calculations for flexibility. The end products of *coverage.py* such as the reference fasta and the sorted BAM files can be used as input into prokaryotic and eukaryotic binning modules analogously to the assembly fasta and sorted BAM file from *assembly.py*.

#### *binning-prokaryotic.py*—Iterative consensus binning for recovering prokaryotic genomes with lineage-specific quality assessment

**Fig. 2:**
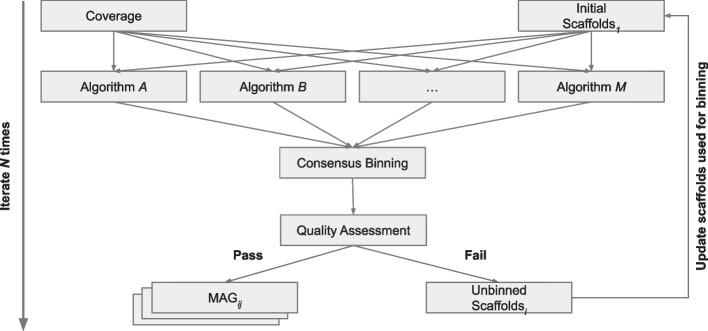
Schematic iterative binning algorithm. *VEBA’s* iterative binning algorithm and the flow of contigs through the procedure

The prokaryotic binning module implements a novel iterative consensus binning procedure that uses *CoverM* (https://github.com/wwood/CoverM) for fast coverage calculations, multiple binning algorithms (*MaxBin2* (marker set = 107); *MaxBin2* (marker set = 40) [33]; *MetaBAT2* [39]; and *CONCOCT* [40]), consensus dereplication and aggregate binning with *DAS Tool* [34], the consensus domain wrapper for *Tiara* [61] for removing eukaryotes at the MAG level, and *CheckM* for quality assessment where poor quality MAGs are removed (e.g., completeness ≤ 50% and/or contamination > 10). The novelty of this procedure is that the unbinned contigs are stored and fed back into the input of the binning procedure using a separate random seed state allowing for an exhaustive, yet effective, approach in extracting high quality and difficult to bin genomes; number of iterations specified by *--n_iter* option (Fig. 2). Gene calls are performed using *Prodigal* [73] and the gene models (GFF3 Format) are modified to include gene and contig identifiers for use with downstream feature counting software. Although *CheckM* can handle CPR, it cannot do so with the recommended *lineage_wf* directly in the current version but instead with a separate manual workflow. The prokaryotic binning module allows for basal bacteria to filter through intermediate genome quality checks, runs *GTDB-Tk* [42] for genome classification, reruns *CheckM* CPR workflow for said genomes, updates the genome set with adjusted completeness and contamination scores, and then filters out genomes that do meet the completeness and contamination cutoffs. The input alignment file is utilized using *featureCounts* to produce counts tables for the gene models and MAGs. Lastly, genome statistics such as N50, number of contigs, and genome size are calculated using *SeqKit.* Utility scripts, installed with *VEBA*, are run in the backend to modify prodigal gene models, consensus domain classification of MAGs using *Tiara* contig predictions, along with several fasta and pre/post-processing scripts. The input to this module is a fasta file (typically the scaffolds.fasta from *metaSPAdes*) and sorted BAM while the output includes the prokaryotic MAGs via *Prodigal*, gene models, identifier mappings, counts tables, *CheckM* output, *GTDB-Tk* output, and unbinned fasta. MAG naming scheme for prokaryotes follows [SampleID]_[Algorithm]_P.[Iteration]_[Name] (e.g., SRR17458623_METABAT2_P.1_bin.1). As *MaxBin2* takes several orders of magnitude longer than *MetaBAT2* and *CONCOCT* when using coverage from multiple samples, there is an option to exclude *MaxBin2* operations in the workflow (i.e., --*skip_maxbin2*).

#### *binning-eukaryotic.py*—Binning for recovering eukaryotic genomes with exon-aware gene modeling and lineage-specific quality assessment

The eukaryotic binning module uses several checks and state-of-the-art software to ensure high quality genomes. In particular, non-prokaryotic-biased binning algorithms *MetaBAT2* [default] (coverage calculated with *CoverM*) or *CONCOCT* (coverage calculated using *CONCOCT* scripts) is used for binning out genomes followed by a genome size filter (2,000,000 bp is the default). *VEBA’s* approach towards eukaryotic binning is to perform domain prediction at the bin level rather than the contig level in order to capture organelles and potentially misclassified contigs. To implement this approach, *VEBA* performs the following operations in the backend: (1) contigs from *MetaBAT2* or *CONCOCT* bins are fed into *Tiara* to produce prediction probability vectors for each contig; (2) prediction probabilities are aggregated with respect to bin assignment to produce logits; (3) logits are transformed into bin-level probabilities using the softmax transformation [default]. Contigs from the eukaryotic MAGs are input into *MetaEuk's* *easy-predict* workflow [51] using our custom consensus microeukaryotic database (see *Database* section in *Methods*). Although *MetaEuk* is a high-quality software suite, the identifiers from *MetaEuk* are very complex, long, and include characters that are often problematic for downstream applications including parsing, file naming systems, and certain programs with simplified identifier requirements such as *Anvi’o* [54]. In addition, the gene model GFF files are not intuitive, compatible with *Prodigal* GFF files or *featureCounts* without major modification. Therefore, we developed an essential wrapper for *MetaEuk* that simplifies identifiers (i.e., [ContigID]_[GeneStart]:[GeneEnd]([strand])), ensuring no duplicates are produced, creates a GFF file that can be concatenated with the *Prodigal* GFF file for use with *featureCounts,* and several identifier mapping tables for seamless conversion between original and modified identifiers. Lineage-specific genome quality estimation is performed using *BUSCO* [56] where poor quality MAGs are removed (e.g., completeness < 50% and contamination > 10). Gene counts are computed using *featureCounts* at the gene level. Lastly, genome statistics such as N50, number of contigs, and genome size are calculated using *SeqKit.* The input to this module is a fasta file (typically the unbinned.fasta from the prokaryotic binning module) and sorted BAM while the output includes the eukaryotic MAGs, gene models via *MetaEuk*, identifier mappings, *BUSCO* output, counts tables, and unbinned fasta. Iterative binning is not currently available for eukaryotic genome recovery as no consensus binning tool is available, therefore, iterative binning would result in diminishing returns. MAG naming scheme for eukaryotes follows [SampleID]_[Algorithm]_E.[Iteration]_[Name] (e.g., ERR2002407_METABAT2_E.1_bin.2).

#### *binning-viral.py*—Detection of viral genomes and quality assessment

Viral binning is performed using *VirFinder* [32] to extract candidate viral contigs (e.g., *P* < 0.05 [default]). The candidate viral contigs are then input into *CheckV* [31] where quality assessment removes poor quality or low confidence viral predictions. The filtering scheme is based on *CheckV* author recommendations [74] in which a candidate viral contig is considered if it meets the following criteria: (1) number of viral genes ≥ 5 × number of host genes; (2) completeness ≥ 50%; (3) *CheckV* quality is either medium-quality, high-quality, or complete; and (4) *MIUViG* quality is either medium-quality, high-quality, or complete [75]. Proviruses can be included by using the *--include_*proviruses flag. After poor quality viral contigs are removed, *Prodigal* is used for gene modeling and *SeqKit* is used for useful genome statistics. The input to this module is a fasta file (typically the unbinned.fasta from the eukaryotic binning module) while the output includes the viral MAGs, gene models via *Prodigal*, identifier mappings, and *CheckV* quality assessment output. Iterative binning is not applicable for viral detection as algorithms are executed on a per-contig basis and all viral genomes will be identified on first pass. MAG naming scheme for viruses follows [SampleID]_[Algorithm]_[Name] (e.g., SRR9668957_VIRFINDER_Virus.1).

#### *classify-prokaryotic.py*—Taxonomic classification and candidate phyla radiation adjusted quality assessment of prokaryotic genomes

The prokaryotic classification module is a useful wrapper around *GTDB-Tk* which either combines the resulting archaea and bacteria summary tables or runs *GTDB-Tk lineage_wf* from the beginning. If genome clusters are provided, then it performs consensus lineage classification.

#### *classify-eukaryotic.py*—Taxonomic classification of eukaryotic genomes

The eukaryotic classification module utilizes the target field of *MetaEuk* gene identifiers and the taxonomic lineage associated with each source genome. The default marker set is *eukaryote_odb10* from *BUSCO* but custom marker sets are support along with the inclusion of all genes not just marker genes. An option to include marker-specific noise cutoff scores is also available using the *--scores_cutoff* parameter which is default behavior with *BUSCO’s eukaryote_odb10* provided noise thresholds. For each MAG, bitscores are accumulated for each taxonomic level and taxonomy is assigned with leniency specified by the leniency parameter with high leniency resulting higher order taxonomic assignments. If genome clusters are provided, then it performs consensus lineage classification.

#### *classify-viral.py*—Taxonomic classification and isolation source of viral genomes

The viral classification module utilizes the *CheckV* database along with the best hit lineage and source habitat information from the *CheckV* output. This includes a look up of *CheckV* identifiers based on direct terminal repeats and *GenBank* identifiers when applicable. If genome clusters are provided, then it performs consensus lineage classification and consensus habitat annotation.

#### *cluster.py*—Species-level clustering of genomes and lineage-specific orthogroup detection

To leverage intra-sample genome analysis in an inter-sample analytical paradigm, genome clustering and lineage-specific orthogroup detection is necessary. The clustering module first uses *FastANI* [76] to compute pairwise ANI and these are used to construct a *NetworkX* graph object where nodes are genomes and edges are ANI values [77]. This graph is converted into subgraphs of connected components whose edges are connected by a particular threshold such as 95% ANI [default] as recommended by the authors for species-level clustering. These species-level clusters (SLC) are then partitioned and *OrthoFinder* [78] is then run on each SLC panproteome. The input is a list of genome paths and list of protein fasta paths while the output includes identifier mappings between genomes, SLCs, contigs, proteins, and orthogroups. The nomenclature preferred by *VEBA* is the PSLC, ESLC, and VSLC for the prefix of each cluster (e.g., *PSCL0*).

#### *annotate.py*—Annotate translated gene calls against NR, Pfam, and KOFAM

Annotation is performed using best-hit annotations and profile HMMs. First proteins are aligned against NCBI non-redundant protein database (other databases are supported) using *Diamond* [79, 80]. After annotation, protein domains are identified using the *Pfam* database [58] via *HMMER* [81] and KEGG orthology is characterized via *KOFAMSCAN* [57].

#### *phylogeny.py*—Construct phylogenetic trees given a marker set

The phylogeny module is a tool used for phylogenetic inference and constructing phylogenetic trees for genomes given a reference marker set (see *Databases* section of *Methods*). This is performed by the following method: (1) identify marker proteins using *HMMSearch* from the *HMMER3* suite; (2) create protein alignments for each marker identified *MUSCLE* [82]; (3) trim the alignments using *ClipKIT* [83]; (4) concatenate the alignments; (5) approximately-maximum-likelihood phylogenetic inference using *FastTree2* [84]; and (6) optional maximum likelihood phylogenetic inference using *IQ-TREE2* [85]*.* An option to include marker-specific noise cutoff scores is also available using the *--scores_cutoff* parameter. Poor-quality genomes that do not meet a threshold in the proportion of markers in the reference are removed using the *--minimum_markers_aligned_*ratio parameter. Similarly, non-informative markers that are not prevalent in the query genomes are removed using the *--minimum_genomes_aligned_ratio* parameter.

#### *index.py*—Build local or global index for genomes

The index module creates reference indices for alignments in both local or global paradigms. In the local paradigm, an index is created for all the assembled genomes concatenated together for each sample. This is useful in situations where perfectly paired metagenomics and metatranscriptomics are available where the metatranscriptomics can be mapped directly to the de novo reference generated from the metagenomics. However, this is not applicable in all cases such as when there is not a perfect overlap between metagenomics and metatranscriptomics where a global paradigm is more appropriate. In the global paradigm, assembled genomes are concatenated across all samples and an alignment index is created for this concatenated reference. Currently, *Bowtie2* [64] is the only alignment software packages supported.

#### *mapping.py*—Align reads to local or global index of genomes

The mapping module uses local or global reference indices generated by the index module and aligns reads using *Bowtie2.* The alignment files are sorted to produce sorted BAM files using *Samtools* which are then indexed*.* Coverage is calculated for contigs via *Samtools* and genome spatial coverage (i.e., ratio of bases covered in genome) is provided. Reads from the sorted BAM files are then fed into *featureCounts* to produce gene-level counts, orthogroup-level counts, MAG-level counts, and SLC-level counts.

### Local and global reference indexing

Multi-omics analyses such as paired metagenomics and metatranscriptomics are becoming increasingly more common to study complex systems. However, the logistics of sampling introduce two main scenarios: (1) a perfect sample overlap between modalities; and (2) an incomplete (or even disjoint) overlap between modalities. To address both scenarios, our software implements both local and global read alignment. In the local paradigm, binned MAGs are concatenated and alignment indexes are generated for each sample. In the global paradigm, all the binned MAGs from all the samples are concatenated and a single index is generated for the concatenated assembly. Local read alignment are limited to scenarios in which there is a perfect overlap of samples between modalities, is less computationally intensive, and has the benefit of decreasing ambiguous mapping events (i.e., mapping equally well to more than one reference). The caveats of local read alignment is that there may be genomes that are present but were not properly binned and will not be accounted for in the final counts table. The benefits of global read alignments is that they can be used for any dataset even if there are no overlapping samples. The caveats of this approach is that it is more computationally expensive and the increased likelihood of ambiguous mapping events; though, the latter is addressed when grouping features by the clustering mentioned prior and summing the counts. Both local and global indexing are implemented using the *index.py* module.

### Hybrid sample-specific and consensus approach to metagenomics

The approach implemented in this software suite is a hybrid of sample-specific and consensus approaches with several rounds of dereplication. The benefits of using consensus metagenomics such as coassembly and metagenomic binning on said assemblies is that they yield biological features (e.g., genes, contigs, genomes, etc.) that are comparable across multiple samples. For example, a coassembly from *N* metagenomic samples will result in a community-level metagenome where the reads can be aligned resulting in contigs that are comparable across all samples. While this approach is convenient from an analytical perspective, it is prone to producing MAGs that are a compilation of multiple strains resulting in more complete composite MAGs rather than sample-specific MAGs more closely representing source strains. Although current NGS-based metagenomics do not allow for in silico recovery of individual organisms without probes, sample-specific approaches result in less complex problems to solve by assembly and binning algorithms than coassembly-based approaches. In addition to producing composite genomes, coassembly-based methodologies use considerably more compute resources during assembly as the *k*-mer space increases. However, coassembly-based binning can have benefits such as the multi-split approach in *VAMB* [86] where assemblies from different samples are merged for binning but then split into individual bins based on each sample; an approach that can be implemented using any non-marker-based binning algorithm with post hoc procedures. For clarification, in this study we define bins as putative genomes output from binning algorithms and MAGs as genomes that have been quality assessed using metrics from *CheckM*, *BUSCO*, or *CheckV* for prokaryotic, eukaryotic, and viral genomes, respectively.

On the contrary, sample-specific metagenomics are more scalable and benefit from less complex computational problems to solve by assembly and binning algorithms as the samples represent a single community instead of a mixture of communities. In addition, the assemblies and the resulting MAGs binned from said assemblies are more biologically accurate as they are not composites based on multiple samples and communities. However, the caveat of pursuing a sample-specific approach is that the resulting biological features are not comparable between samples. For example, metagenomes *A, B,* and *C* all have their own assemblies with their own disjoint set of contigs that comprise a disjoint set of MAGs so the reads used to assemble contigs in *A* are not used to assemble contigs in *B* or *C*. One approach would be aligning reads directly to each respective sample but this would produce an inherently sparse concatenated matrix when concatenating counts tables. Another alternative would be aligning reads to a concatenated assembly but—due to the likelihood of similar but distinct strains of the same species occurring in multiple samples—reads will either be randomly assigned or multi-mapped. The former would result in another sparse matrix and latter in a multi-mapped counts table both of which violate assumptions of compositional data analysis [87] with the latter known to introduce downstream analytical complications [88–92]. Further, sample-specific and consensus metagenomics is analogous to amplicon-sequence variants [21, 93] and operational taxonomic units [22] in that MAGs yielded by the former can be added to existing databases as their construction is not dependent on multiple samples. Although this approach prioritizes sample-specific binning, it also supports multi-sample binning, introducing the concept of a pseudo-coassembly, which we prefer to implement when using all the unbinned contigs from the assemblies within a dataset as none of the samples alone have complete genomes. We define pseudo-coassembly as the union of contigs from all samples within a dataset that could not be binned using sample-specific binning approaches with the premise that the genomes are present in each sample but could not be resolved due to biological, technical, or computational limitations. The approach to implementing hybrid sample-specific and consensus approaches synergistically in this study is to use dereplication of sample-specific metagenomics via clustering.  In addition to pseudo-coassembly binning, *VEBA* also supports workflows for *bona fide* coassembly and subsequent binning.

### Iterative binning

Most metagenomic genome binning pipelines are not exhaustive nor are they iterative in the sense that unbinned contigs are fed back into the algorithm. While this may suffice for metagenomic samples of low to mid-level complexity, a one-and-done approach is not effective in maximizing the available information content hidden within mid-to-high level complexity metagenomes. Further, genomes that may be problematic for binning algorithms to extract on a first pass may be less problematic in subsequent runs. While running a single binning algorithm iteratively is useful, the benefits are magnified when using the results of multiple binning algorithms (e.g., *MetaBAT2*, *MaxBin2*, and *CONCOCT*) followed by dereplication tools (e.g*., DAS Tool*) referred to as consensus binning and as has been benchmarked extensively in prior research [29, 34]. Consensus binning is a powerful approach as it uses the strengths and bypassing the weaknesses of each binning algorithm to produce a single combination of bins based on the individual binning algorithms; some of which could not have been identified alone by any single algorithm. While *VEBA* does not specifically introduce a unique binning algorithm, it uses a combination of consensus binning, alternative random seed states, and iterative binning of unbinned contigs the prokaryotic binning in a unique workflow that can be adapted to incorporate other software packages.

To further complement iterative sample-specific binning procedures, the unbinned contigs from prokaryotic, eukaryotic, or viral binning methods can be aggregated into a pseudo-coassembly with a post hoc binning based on concatenated contigs containing incomplete genomes. This post hoc pseudo-coassembly binning is optional and available for users to maximize usage on all the available data if desired. The logic for this procedure is that genomes present in each individual sample are incomplete and fragmented which is why they were not recovered during the sample-specific binning and pseudo-coassembly binning has the potential to combine said fragments into a complete genome with reduced likelihood of contaminated genomes than binning using the entire coassembled dataset. The schematic for the iterative binning algorithm is shown in Fig. 2. Iterative binning is currently not implemented for eukaryotes because there is not yet an analog to *DAS Tool* for the eukaryotic domain.

### Clustering in genomic and functional space

*VEBA* clusters in both genomic and functional space. More specifically, clustering strains into species-level clusters (SLC) and proteins into SLC-specific orthogroups (SSO). Clustering genomes into SLCs have been successfully implemented in the past when dereplicating genomes from different assemblies [94] using average nucleotide identity (ANI). In this implementation, we use 95% ANI to cluster genomes of the same species from different genomes to produce SLCs but this parameter can be adjusted. We extend this logic to functional space by using SLC-specific orthogroup (SSO) analysis on all open reading frames (ORF) to yield functional genes that are representatives of specific proteins within a taxonomic grouping (e.g., species) in a dataset. Genome and protein-level clustering into SLCs and SSOs, respectively, allows the user to conduct analysis using biological features that are directly comparable across samples while operating under the constraints of compositional data analysis assumptions. Both genomic and functional clustering are performed using the *cluster.py* module.

### Genomic and functional feature compression for dimensionality reduction

Many downstream metagenomics methods require statistical analysis, either classical or machine-based, to model a system and explore a particular hypothesis. Using metagenomics datasets to model complex phenomena such as clinical phenotypes or ecological disturbances can be extremely difficult due to the vast number of features relative to the number of samples. When the number of features (e.g., MAGs and ORFs) greatly exceeds the number of observations (e.g., biological sample), the likelihood of statistical anomalies increases due to the “curse of dimensionality” [95]. Feature compression is a feature engineering method that aggregates the values of features with respect to specific groupings and can be used to reduce the dimensionality of the data and, therefore, minimize anomalous phenomena. To compress biological features for counts tables, *VEBA* utilizes the SLC and SSO clustering to aggregate the read counts from the *mapping.py* module by summing the counts for each original feature with respect to their clustered grouping. For instance, given a mapping of 1000 ORFs to 100 SSOs, an ORFs counts matrix of dimensionality (*N*_*Samples*_ = 80, *M*_*ORFs*_ = 1000) is aggregated to a dimensionality (*N*_*Samples*_ = 80, *M*_*SSOs*_ = 100).

The feature compression ratio (FCR) is an informative heuristic that can not only provide information on how much the dimensionality has been reduced but also on how complex a community is in terms of redundancy in organisms and functionality. The FCR is calculated as 1 − *N*_*Clusters*_*/N*_*Features*_ where *N*_*Clusters*_ is the number of clustered features and *N*_*Features*_ is the number of original features. For example, if there are 200 MAGs that collapse into 50 SLCs then the FCR is 1 − (50/200) = 0.75 which is interpreted as SLCs encode roughly the same information content in 75% fewer dimensions. The operation is the same for functional feature aggregation of ORFs into SSOs with the one distinction being that only clustered ORFs are considered. Modifying an earlier example, if there were 1100 ORFs in total with 1000 ORFs clustered into 100 SSOs then the functional FCR would be 1 − (100/1000) = 0.9 or 90%. Functional FCRs can be interpreted as the functional information in all clustered proteins can be represented in 90% fewer features. While this feature compression may not be suitable for granular analysis that investigates strain-level or isoform-level properties, it applies to the vast majority of studies where species and their associated functionalities are the focus.

### Phylogenetic inference of recovered diatom genomes

Phylogenetic inference of diatom genomes recovered from *Plastisphere* was performed using the *phylogeny.py* module with *eukaryote_odb10* marker set and the associated noise cutoffs from *BUSCO.* Proteomes from related diatoms from *VEBA*’s microeukaryotic protein database including *MMETSP* and *NCBI* were included in inference for placement. A threshold of 0.95 was used for *--minimum_genomes_aligned_ratio* to remove poor quality genomes. A threshold of 0.2 was used for *--minimum_markers_aligned_ratio* to remove non-informative markers. Phylogenetic trees were visualized using *ETE* in Python.

### Differential co-occurrence networks and compositional data analysis

Network analysis was performed on the *Plastisphere* dataset using read counts from the *mapping.py* module and a global index from the *index.py* module. In short, reads were mapped and read alignments were counted with respect to contigs using the *mapping.py* module. Aggregating contig counts instead of ORF counts is more accurate in abundance-based approaches because it accounts for genes missed by gene modeling algorithms and reads that land between coding regions. The contig-level counts are aggregated by MAGs and then by SLCs to reduce dimensionality, compress strains into species, and yield taxonomic features that are both compositionally-valid and comparable across samples. This aggregation is performed using *merge_contig_mapping.py* utility script.

For interpretation and visualization, counts from the SLC features were further aggregated in a domain-specific manner. More specifically, there were far more prokaryotic and viral SLCs than eukaryotic SLCs so we grouped prokaryotes by their genus-level taxonomy and viruses by their VOG classification (*Retrovirales* or *Caudovirales*). This aggregate feature matrix was then filtered by removing features that are in less than 40% of the samples.

Networks were implemented using the following approach: (1) split feature matrix into (1a) mature plastic biofilm samples and (1b) early plastic biofilm samples; (2) *ρ* proportionality for ensemble co-occurrence of *Network*_*Mature*_ and *Network*_*Early*_ separately [87, 88, 91, 92] using the *EnsembleNetworkX Python* package [96] with 1000 iterations; (3) compute differential connectivity via *Network*_*Mature*_*—Network*_*Early*_; (4) consider only edges that have positive associations in both conditions (negative *ρ* associations are non-trivial to interpret) and have a differential connectivity of at least 0.1; and (5) hive plot of differential connectivity edges implemented via *Hive NetworkX* [97]*.* Network analysis was performed only on the *Plastisphere* dataset as this had several taxa for each domain which was not the case in *MarineAerosol* or *Netherton* datasets.

Clustered abundance heatmaps were implemented using the following approach: (1) Center Log-Ratio (CLR) transformed counts with pseudo-count of *1/m*^2^ where *m* indicates number of features; (2) Aitchison distance hierarchical clustering for samples; (3) *ρ* dissimilarity hierarchical clustering for features; and (4) heatmap via *Seaborn Python* package [98]. Hierarchical clustering was performed using average linkage implemented and visualized using the *Agglomerative* class of the *Soothsayer* Python package [9, 99]. Dissimilarity representation of the *ρ* proportionality calculated via 1—*ρ* as implemented in correlation distance calculations of *SciPy* [100]. Aitchison distance is calculated via Euclidean distance on CLR-transformed counts.

## Results and discussion

### A walkthrough of *VEBA*

*VEBA* is a modular software suite that supports users at different stages of metagenomics analysis such as starting from reads, contigs, proteins, or MAGs. The workflows are designed for sample-specific metagenomics followed by a post hoc multi-sample approach via a pseudo-coassembly to merge incomplete and fragmented genomes from different samples (Fig. 1). In addition, the design of *VEBA* allows for purely sample-specific or *bona fide* coassembly approaches as well.

*VEBA* supports complete end-to-end metagenomics workflows from reads all the way up to fully annotated and clustered MAGs. In a complete end-to-end metagenomics workflow, users starting with raw reads would input fastq formatted reads into the *preprocess.py* module which performs trimming/adapter removal, an optional decontamination based on a reference genome (e.g., human), an optional k-mer based removal/quantification (e.g., ribokmers), read pairing to ensure each forward read has a reverse counterpart (essential for *SPAdes*-based assemblers), and read statistics are calculated for each stage for a full accounting of reads. Cleaned reads are input into the *assembly.py* module where reads are assembled using *SPAdes*-based assemblers (e.g., *metaSPAdes*), reads are mapped to the assembly to produce a sorted BAM file, counts tables are generated, and assembly statistics are calculated. Assembled contigs and the sorted BAM file from the *assembly.py* are then input into the *binning-prokaryotic.py* module where iterative consensus binning is performed using *MetaBAT2*, *CONCOCT*, and an optional *MaxBin2* (using 2 separate marker sets) followed by *DAS Tool* for consensus binning (Fig. 2), gene modeling using *Prodigal,* quality assessment with *CheckM*, phylogenetic inference with *GTDB-Tk* after all iterations are complete to adjust quality for CPR using the appropriate lineage marker set, and ORF-level counts table are compiled. The unbinned contigs from the *binning-prokaryotic.py* module and the sorted BAM file are used as input into the *binning-eukaryotic.py* module, which bins genomes using either *MetaBAT2* or CONCOCT, predicts whether or not bins are eukaryotic using *Tiara,* models genes using *MetaEuk* with the *VEBA* microeukaryotic protein database, quality assesses genomes using *BUSCO,* and ORF-level counts table are compiled. The unbinned contigs from the *binning-eukaryotic.py* module are input into the *binning-viral.py* module where *VirFinder* is used to identify candidate viral contigs, quality is assessed using *CheckV,* and models genes using *Prodigal.* A sorted BAM file is not required but if provided then ORF-level counts table are compiled. If the user desires to strictly implement a sample-specific workflow then the next steps pertaining to pseudo-coassembly binning can be skipped but to effectively extract as much information as possible from a dataset then the pseudo-coassembly steps are recommended for datasets that contain samples with highly similar biological sources. For pseudo-coassembly binning, the user concatenates unbinned contigs from all assemblies (available in the output directories) into a pseudo-coassembly fasta file, the *coverage.py* module aligns reads from each sample to provide sorted BAM files based on this multi-sample reference, sorted BAM files are used to create a contig-level counts table, and sequence statistics are calculated. This pseudo-coassembly reference fasta and the associated sorted BAM files are then used as input into the *binnning-prokaryotic.py* module with the unbinned contigs getting sent to a final round of *binning-eukaryotic.py*. None of the pseudo-coassembly gets reinput into the *binning-viral.py* because the backend algorithms work on the contig-level and all high-quality viruses have already been recovered. Once the genome binning is complete, clustering of genomes into SLCs and proteins into SSOs from each domain is performed using the *cluster.py* module which also generates identifier mappings used to reference between contigs, MAGs, SLCs, ORFs, and SSOs. Next, reads are mapped to either local or global references using the *index.py* and *mapping.py* modules to compile contig and ORF-level counts tables. Counts tables are then aggregated using the clustering from *cluster.py* for MAGs and ORFs to engineer SLC and SSO features, respectively, and compute their feature compression ratios (FCR) to quantify the dimensionality reduced for genomic FCR (1 − *N*_*SLC*_*/N*_*MAG*_) and functional FCR (1 − *N*_*SSO*_*/N*_*ORF*_)_*.*_ Genomes from each domain are classified using the *classify-prokaryotic.py, classify-eukaryotic.py,* and *classify-viral.py* modules which uses *GTDB-Tk, MetaEuk*, and *CheckV* results, respectively. Genes are annotated using NCBI’s non-redundant, *Pfam*, and *KOFAM* databases with the *annotate.py* module. Finally, phylogenetic trees are inferred using the *phylogeny.py* module with either custom marker sets or *VEBA* provided marker sets.

Another end-to-end workflow would be recovering and annotating RNA viruses in metatranscriptomes. If reads are provided as input then reads are cleaned with *preprocess.py* just as in the metagenomics workflow previously and assembled into transcripts via *rnaSPAdes* in the *assembly.py* module. If transcripts were assembled separately (e.g., *Trinity* [101]) then these transcripts can be provided instead. Viruses are then recovered from the de novo transcripts with the *binning-viral.py* module and classification of viruses is performed using the *classify-viral.py* module. This modularity extends to other domain-specific workflows and can include or omit counts table generation, gene annotations, and phylogenetic analysis.

As mentioned, *VEBA* is modular so users could use the suite to cluster existing genomes that they have downloaded or binned using custom methods, annotate existing gene models or protein sets, build phylogenetic trees from existing genomes, or map reads to existing references. A user can even skip a domain or run in non-iterative mode if desired. Further, users can use *VEBA’s* microeukaryotic protein database to model genes and phylogenetically characterize genomes not derived from *VEBA*. *VEBA* maximizes the input/output of modules to increase the productivity of users and their metagenomics workflow. For instance, whenever sequences are generated, they come with sequence statistics or when BAM files are used as input they come out with counts tables to name a few examples. Please refer to the *Methods* section for a more detailed explanation of each module and the walkthroughs available on GitHub for more workflows.

### Microeukaryotic protein database

A protein database is required not only for eukaryotic gene calls using *MetaEuk* and these results can also be leveraged for MAG annotation. Many eukaryotic protein databases exist such as *MMETSP*, *EukZoo*, and *EukProt*, yet these are limited to marine environments, include prokaryotic sequences, or include eukaryotic sequences for organisms that would not be expected to be binned out of metagenomes such as metazoans. While it may be possible to bin fragments of higher eukaryotic genomes, this is often not the objective of many metagenomic studies where microorganisms are the focus. We combined and dereplicated *MMETSP*, *EukZoo*, *EukProt*, and *NCBI* non-redundant to include only microeukaryotes such as protists and fungi. This optimized microeukaryotic database ensures that only eukaryotic exons expected to be represented in metagenomes are utilized for eukaryotic gene modeling and the resulting *MetaEuk* reference targets are used for eukaryotic MAG classification. This microeukaryotic targeted protein database lowers the database size and computational resources needed for eukaryotic gene modeling and classification than including additional prokaryotic or metazoan proteins. *VEBA’s* microeukaryotic protein database includes 48,006,918 proteins from 42,922 microeukaryotic strains (Table 1).

**Table 1:** Microeukaryotic protein database taxonomy synopsis

	Number Representatives				Number of sequences
Class	Order	Family	Genus	Species	
Aconoidasida	*2*	*5*	*12*	3366	420945
Agaricomycetes	20	122	730	7633	3598622
Arthoniomycetes	*1*	*6*	*71*	277	557
Bacillariophyceae	25	49	139	1139	3695969
Bangiophyceae	3	4	27	298	91032
Conoidasida	3	12	26	548	283655
Coscinodiscophyceae	11	24	49	369	761079
Cryptophyceae	5	9	18	126	1281699
Dinophyceae	13	37	80	404	9452835
Dothideomycetes	33	120	796	4173	2193726
Eumycetozoa	7	17	48	212	110038
Eurotiomycetes	10	29	137	2028	2406417
Florideophyceae	28	95	650	4014	140811
Fragilariophyceae	9	12	62	216	226623
Glomeromycetes	4	10	30	126	456928
Haptophyta	8	15	31	96	847085
Kinetoplastea	4	4	28	355	511789
Lecanoromycetes	15	66	435	2593	103042
Leotiomycetes	9	32	215	795	737176
Mediophyceae	8	10	49	155	190677
Microbotryomycetes	5	7	15	107	121936
Mucoromycetes	1	14	52	184	583544
Oligohymenophorea	10	37	70	406	266349
Pezizomycetes	1	15	143	709	224226
Phaeophyceae	12	43	236	1244	58542
Pucciniomycetes	5	19	62	379	228062
Saccharomycetes	1	15	83	844	1157942
Sordariomycetes	31	99	705	7228	3772436
Spirotrichea	8	34	84	199	429742
Tremellomycetes	4	17	50	316	377309
Ustilaginomycetes	4	10	25	169	137101
Xanthophyceae	4	11	21	149	49722
Other (N = 147 classes)	242	346	663	2065	13089302
Total classes = 179	546	1345	5842	42922	48006918

### Case study I: The “Plastisphere” microbiome of early and mature plastic biofilm communities

The *Plastisphere* microbiome (BioProject: PRJNA777294, *N* = 44 metagenomic samples, 237 gigabases) is a dataset that includes environmental microbial communities from early and mature stage biofilms formed on macroplastics in a marine environment [102] (Additional file 1: Table S1). Around 5–11% of annual plastic production is input into the ocean each year [103, 104] and researchers predict these plastics may last hundreds to thousands of years because of their stability and durability [105]. As the rate of plastic input into the ocean greatly exceeds the degradation rate, the accumulation of plastic and microplastics in the food chain presents itself as an unprecedented threat not only to ecological health but public health, while also being a new anthropogenically introduced habitat. Studies have shown that microplastics can transmit protozoan pathogens [106], induce reproductive toxicity [107] and are not uncommon in the human body [108] including reproductive organs such as the placenta [109]. The premise of *Bos *et al*. 2022* was to identify and characterize emergent marine microbial biofilm communities during both the early and late stages of plastic colonization using natural seawater communities as the seed.

The bacterial microbiome was previously characterized using coassembly-based genome binning and a strict quality threshold to yield only high-quality genomes (completeness ≥ 70 and contamination < 2). In the original study, 37 high-quality MAGs, including 14 *Alteromonas*, 4 *Marinobacter*, and 8 *Marisediminitalea* MAGs were recovered from early colonization incubations. Using the same genome quality thresholds as the original study, our iterative method was able to isolate 92 high-quality bacterial MAGs, including a novel species of *Gracilibacteria* from the UBA6489 genus, as well as 3 diatom and 1 pelagophyte eukaryotic MAGs. These eukaryotic MAGs also include a *Chrysoreinhardia* sp strain CCMP3193 and a novel *Bacillariophyceae* diatom genera both of which lack genome representatives in any public database*.* In addition to prokaryotes and eukaryotes, we were able to isolate 119 high-quality viral MAGs (clustering into 81 SLCs with 1,317 genes) including 71 *Retrovirales*, 6 *Caudovirales*, 3 *Inoviridae*, 1 *CressDNAParvo*, and 35 uncharacterized viruses*.*

**Fig. 3:**
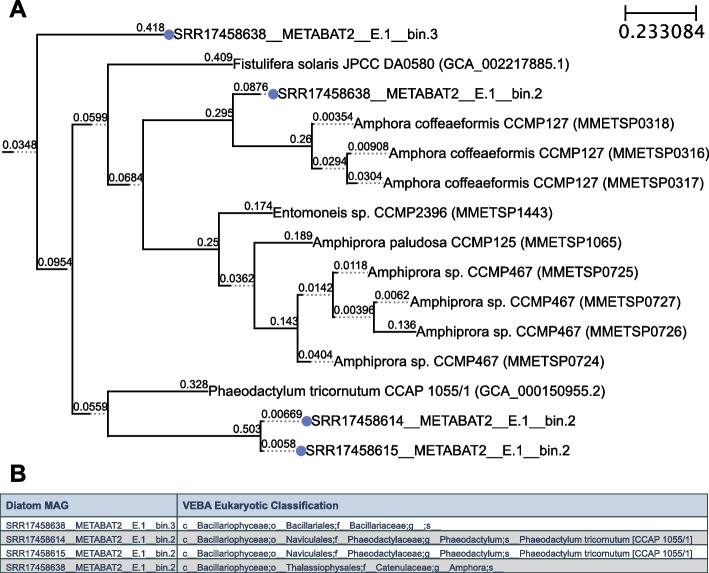
Phylogenetic inference of diatoms recovered in Plastisphere. **A** Phylogenetic tree using the concatenated alignment of *eukaryote_odb10* marker set from *BUSCO* and *FastTree2* visualized with *ETE 3*. **B** *VEBA* eukaryotic classifications for diatom MAGs

There is information to be gained in medium-quality MAGs, therefore we conducted a secondary analysis with our default operating threshold (completeness ≥ 50 and contamination < 10) where we obtain 127 more medium-quality bacterial MAGs (total of 219 prokaryotic MAGs clustering into 137 SLCs with 1,029,466 genes). In addition to more prokaryotic MAGs, these thresholds yielded an unclassified *Amphora* (diatom) MAG (total of 5 eukaryotic MAGs clustering into 4 SLCs with 78,750 genes); the genus *Amphora* does not have a genome published in any public databases. Phylogenetic inference of *Plastisphere* diatoms agrees with *VEBA*’s eukaryotic classification (Fig. 3). Concatenating unbinned contigs from sample-specific prokaryotic, eukaryotic, and viral binning into a pseudo-coassembly and binning this pseudo-coassembly resulted in additional 25 prokaryotic MAGs but no additional eukaryotic MAGs (Table 2). Of the 219 prokaryotic genomes recovered using *VEBA’s* iterative binning module, the overwhelming majority were represented by *Alphaproteobacteria* (44%), *Gammaproteobacteria* (29%), and *Bacteroidia* (12%) with 168 genomes of novel species. Recovered genome statistics and taxonomy of genomes are detailed in Tables 2, 4, Additional file 3: Table S3.

The genomic FCR was modest for prokaryotes, eukaryotes, and viruses with a percent decrease in dimensionality of 29.7%, 20%, and 32.9% respectively. The functional FCR had a similar trend for prokaryotes, eukaryotes, and viruses with a percent decrease in dimensionality of 31.5%, 18.6%, and 46.4%, respectively (Table 2).

**Table 2:** Genome binning, clustering results, and complexity analysis for case studies

	*Plastisphere*	*MarineAerosol*	*Netherton*
BioProject	PRJNA777294	PRJEB20421	PRJNA551026
Original Study	*Bos *et al*. 2022*	*Michaud *et al*. 2017*	*Williams *et al*. 2020*
Number of samples	44	64	17
Gigabases	237	90	9
**Prokaryotic**			
MAGs (Original Study)	37	8	0
MAGs (Sample-specific)	194(91)^c^	214	15
MAGs (Multi-sample)^b^	25(1)^c^	3	5
MAGs (Total)	219	217	20
SLCs	154	48	12
ORFs	735406	652008	50711
ORFs^a^	706092	615479	47954
SSOs	483864	140638	25848
Genomic FCR	0.296803653	0.778801843	0.4
Functional FCR^a^	0.314729525	0.771498296	0.460983442
**Eukaryotic**			
MAGs (Original Study)	0	17^d^	0
MAGs (Sample-specific)	5(4)^c^	3	0
MAGs (Multi-sample)^b^	0	0	0
MAGs (Total)	5	3	0
SLCs	4	1	Not applicable
ORFs	78750	49958	Not applicable
ORFs (Orthogroups)^a^	78171	46709	Not applicable
SSOs	63661	15335	Not applicable
Genomic FCR	0.2	0.666666667	Not applicable
Functional FCR^a^	0.185618708	0.671690681	Not applicable
**Viral**			
MAGs (Original Study)	0	6^d^	0
MAGs (Sample-specific)	119	345	18
MAGs (Multi-sample)^b^	Not applicable	Not applicable	Not applicable
MAGs (Total)	119	345	18
SLCs	81	69	12
ORFs	1317	20519	602
ORFs (Orthogroups)^a^	1279	20397	598
SSOs	686	3436	393
Genomic FCR	0.319327731	0.8	0.333333333
Functional FCR^a^	0.463643471	0.831543854	0.342809365

^a^Only includes ORFs that are in SSOs

^b^Multi-sample binning uses unbinned contigs from all of the samples in a pseudo-coassembly

^c^Parenthesis indicate completeness ≥ 70 and contamination < 2 as used in original study. Outer indicates completeness ≥ 50 and contamination < 10

^d^Quality was not assessed in original study

**Table 3:** Per iteration genome binning yields

Origin type	Iteration	*Plastisphere*	*MarineAerosol*	*Netherton*
Sample-specific	1	175	202	15
	2	14	7	0
	3	1	4	0
	4	1	1	0
	5	1	0	0
	6	0	0	0
	7	2	0	0
	8	0	0	0
	9	0	0	0
	10	0	0	0
Multi-sample	1	14	3	5
	2	1	0	2
	3	3	0	0
	4	5	0	0
	5	1	0	0
	6	1	0	0
	7	0	0	0
	8	0	0	0
	9	0	0	0
	10	0	0	0
Total	-	219	217	22

**Table 4:** Taxonomy of recovered genomes

Domain	Taxonomy	*Plastisphere*	*MarineAerosol*	*Netherton*
Eukaryotic	c_Bacillariophyceae	4	0	0
	c_Coscinodiscophyceae	0	3	0
	c_Pelagophyceae	1	0	0
Prokaryotic	c_Acidimicrobiia	4	0	0
	c_Actinomycetia	1	0	8
	c_Alphaproteobacteria	97	95	0
	c_Anaerolineae	1	0	0
	c_Babeliae	0	7	0
	c_Bacilli	0	0	13
	c_Bacteriovoracia	2	0	0
	c_Bacteroidia	26	38	1
	c_Chlamydiia	0	2	0
	c_Cyanobacteriia	15	0	0
	c_Gammaproteobacteria	64	70	0
	c_Gracilibacteria	1	0	0
	c_Planctomycetes	4	0	0
	c_Thermoanaerobaculia	1	0	0
	c_UBA1135	0	5	0
	c_Vampirovibrionia	1	0	0
	c_Verrucomicrobiae	2	0	0
Viral	Caudovirales	6	298	8
	CressDNAParvo	1	0	1
	Inoviridae	3	0	0
	PolyoPapillo	0	0	1
	Retrovirales	71	13	0
	Uncharacterized	35	28	8

### Case study II: Ocean–atmosphere aerosolization mesocosm microbiome

The *MarineAerosol* microbiome (BioProject: PRJEB20421, *N* = 64 metagenomic samples, 90 gigabases) is a dataset investigating ocean–atmosphere aerosolization mesocosms and includes environmental microbial communities in ocean water collected before, during, and after an algal bloom using the *Wave Flume* ocean simulator [110] (Additional file 1: Table S1). The types of ocean water included in this study were bulk, surface, and aerosolized sea water. Aerosolized bacteria can travel as far as 11,000 km over the span of days to weeks [111, 112] while algal viruses can remain infectious over several hundred km [113]. Further, airborne microbes and viruses influence climate by seeding cloud formation and inducing ice nucleation [114]. From a clinical setting, airborne microorganisms impact air quality through transmission of allergens [115] and transmit pandemic-scale pathogens such as SARS-CoV-2 [116]. The premise of this study was to identify and characterize the microbial communities in the bulk and surface ocean that were able to effectively aerosolize into the atmosphere.

The original study broadly assessed both singleton genomes and pangenomes of varying quality in addition to read-based taxonomic profiling via *Kraken* [117]. Regarding the assembly-centric metagenomics, the supplementary information reported 8 draft singleton bacterial genomes annotated as basal *Roseobacter*, basal *Proteobacteria*, *Methylophaga*, and *Escherichia coli* along with 17 draft genomes labeled as pangenomes representing diatom fragments, various phages, and several bacterial phyla. These draft genomes were quality assessed by ensuring each genome covered at least 1% of the available reference genome for the closest representative yielding 14 MAGs used in the main study.

Our iterative prokaryotic binning module recovered 217 MAGs clustering into 48 SLCs with 652,008 genes. The overwhelming majority of prokaryotic MAGs represented by *Alphaproteobacteria* (44%), *Gammaproteobacteria* (32%), and *Bacteroidia* (18%) including 162 MAGs representing novel species of *Alphaproteobacteria*, *Babeliae, Bacteroidia*, *Chlamydiia, Gammaproteobacteria,* and *UBA1135* (Table 4, Additional file 3: Table S3). The eukaryotic binning module recovered 3 strains of *Cyclotella meneghiniana,* clustering into 1 SLC with 49,958 genes, which does not have a representative species genome and only one reference genome (*Cyclotella cryptica* CCMP332) available for the entire genera. The viral binning module recovered 345 MAGs that clustered into 69 SLCs with 20,519 genes represented by majority *Caudovirales* (86%) and *Retrovirales* (4%) with the remainder being unclassified viral lineages. This study contained a considerable amount of viral MAGs compared to the other case studies analyzed as expected from the original study’s finding of substantial numbers of reads mapping to existing viral genomes. Recovered genome statistics and taxonomy of genomes are detailed in Tables 2, 4, Additional file 3: Table S3.

The genomic FCR was high across all domains with a percent decrease in dimensionality for prokaryotes, eukaryotes, and viruses of 77.9%, 66.7%, and 80%, respectively. This high FCR essentially means that we captured many strain variants of a smaller subset of species, as defined at the nucleotide identity in a genome scale alignment. As this was a longitudinal experiment with a confined population, it is possible that these strain variants were emergent over the course of the 365-day experiment or were differential abundance over the course of the two phytoplankton bloom cycles. The functional FCR had a similar trend for prokaryotes, eukaryotes, and viruses with percent decreases in dimensionality of 77.2%, 67.2%, and 83.2%, respectively (Table 2).

### Case study III: The Netherton syndrome microbiome

The *Netherton* microbiome (BioProject: PRJNA551026, *N* = 17 metagenomic samples, 9 gigabases) is a dataset that includes human skin microbiome samples from healthy controls and individuals exhibiting Netherton syndrome [118] (Additional file 1: Table S1). Netherton syndrome is rare, multisystemic, autosomal recessive disease [119]. The prognosis of Netherton syndrome may be severe, with significant mortality in early years of life due to potentially fatal complications. Skin and hair defects persist throughout life, but the disorder usually becomes more manageable with age [120]. The pathogenesis of the disease is complex involving interactions between the host immune system and host microbiome, such as the excess microbial proteolytic activity in the setting of LEKTI-1 [121]; there are no specific therapies currently available for patients with Netherton syndrome.

The original study utilized assembly-based metagenomics to focus on virulence-markers from 14 strains of *Staphylococcus aureus,* 8 strains of *Staphylococcus epidermidis*, but recovery of genomes from metagenomes was not a focus of that study. A challenge with skin is that the bulk (> 90%) of the sequencing reads are from the host, thus, the majority of studies only use read-based approaches. Our iterative prokaryotic binning module yielded 20 MAGs clustering into 12 SLCs with 50,711 genes, with species from *Bacilli* (59%), *Actinomycetia* (36%), and *Bacteroidia* (5%). Our analysis recovered genomes for multiple strains of *Staphylococcus aureus* (N = 3 MAGs), *Staphylococcus epidermidis* (N = 2 MAGs), *Staphylococcus pettenkoferi* (N = 3 MAGs), *Staphylococcus caprae* (N = 3 MAGs), and *Staphylococcus capitis* (N = 1 MAG). The eukaryotic binning module was not able to recover any eukaryotic genomes either due to lack of biological material or sequencing depth. The viral binning module recovered 18 MAGs that clustered into 12 SLCs with 602 genes represented by majority *Caudovirales* (44%) along with a *CressDNAParvo*, *PolyoPapillo*, and several unclassified viral lineages. Recovered genome statistics and taxonomy of genomes are detailed in Tables 2, 4, Additional file 3: Table S3. Previous research have linked phages with *Staphylococcus aureus* host evolution and are believed to play major roles in species diversification of staphylococci in general [122], and the co-recovery of putative staph bacteriophage and *Staphylococcus* genomes would be a first in skin microbiome research.

The genomic FCR was modest across all recovered domains with a percent decrease in dimensionality of 40% for prokaryotes and 33.3% for viruses. The functional FCR had a similar trend with 46.1% for prokaryotes and 34.3% for viruses (Table 2).

### Recovered metagenome–assembled genomes

**Fig. 4:**
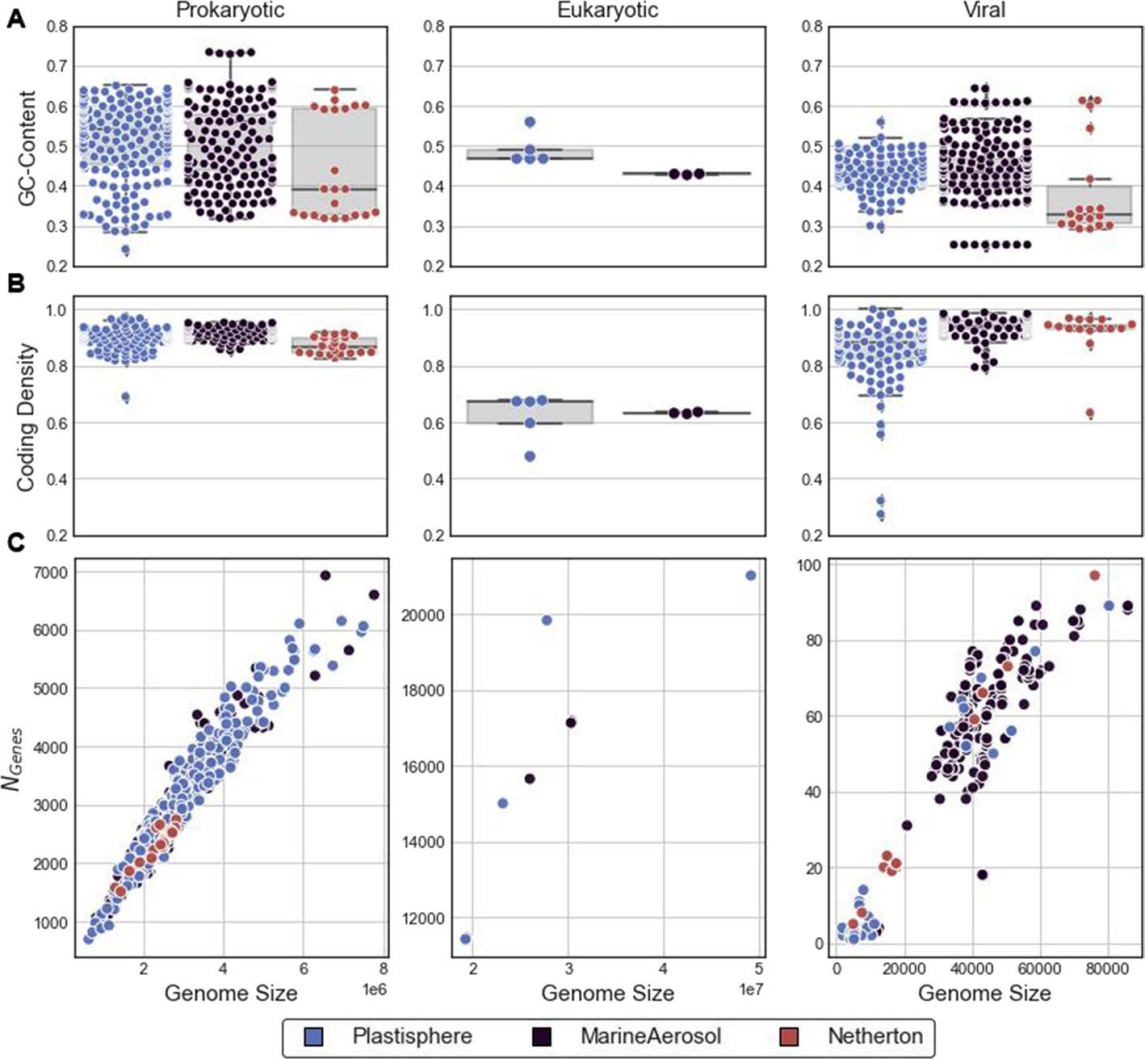
Genome statistics of prokaryotic, eukaryotic, and viral genomes. **A** GC-content and **B** coding-density for prokaryotic, eukaryotic, and viral MAGs for *Plastisphere* (blue), *MarineAerosol* (black), and *Netherton* (red) datasets, respectively. **C**) Relationship between genome size and the number of genes for each MAG

*VEBA* recovered a total of 942 medium-to-high quality MAGs that were detected between the 3 datasets (*N* = 125 samples) which includes 458 prokaryotic, 8 eukaryotic, and 482 viral MAGs. Iterative binning recovered more genomes than non-iterative binning for prokaryotes in complex communities such as the *Plastisphere* and *MarineAerosol* datasets as shown in Table 3; non-iterative binning being only iteration 1 with bins recovered in additional iterations demonstrating the utility of *VEBA’s* iterative binning procedure. As a sanity check, we analyzed the GC-content, coding-density, and distribution of genes relative to the genome size (Fig. 4) to compare with previous research. Most of the prokaryotes had GC-content distributed between 30%—65% across all 3 datasets with the exception of 5 *Planctomycetota* MAGs (i.e., the entirety of *MarineAerosol PSCL10*) that had GC-content ~ 74%; a group that has been previously characterized with high GC-content [123]. In the *Netherton* dataset, we observed 4 *Caudovirales* MAGs and 8 *Corynebacterium* MAGs that have higher than average GC-content (~ 60%) compared to the rest of the MAGs in the dataset which potentially indicates a viral/host pair as phages replicate within their host and often share similar GC-content [124]. In the *MarineAerosol* dataset, we observed 8 uncharacterized viral MAGs from *VSLC8* which contained some of the largest viral genomes (~ 86,000 bp) and lowest GC-content (25%) across all datasets.

We observed a strong relationship between genome size and the number of genes called for each MAG and this trend was consistent across domains for all datasets with very few outliers. For viral outliers, we observed 5 MAGs (i.e., the entirety of *Plastisphere VSLC19*) recovered from 5 separate samples that had no known classification and noticeably fewer genes relative to its genome size compared to the other viral genomes. For eukaryotic outliers, we observed 1 MAG representing *Chrysoreinhardia sp CCMP3193* that had a higher number of genes relative to the genome size. With regards to coding-density, we observed a high number of genes relative to genome size for prokaryotic MAGs relative to eukaryotic MAGs, as expected, where the latter contains introns and more non-coding regions. An uncharacterized species of *Trichodesmium* from the *Plastisphere* dataset had much lower coding-density than all the other prokaryotic MAGs across the 3 datasets; low coding-density in *Trichodesmium* has been documented previously [125]. Note, the presence of *Trichodesmium* on plastic pollution has not been previously reported. Viruses had relatively high coding-density with the exception of a few uncharacterized viral MAGs in the *Plastisphere* dataset (~ 30% compared to the dataset average of 86%) along with a *CressDNAParvo* MAG from *Netherton* dataset (63% compared to the dataset average of 92%).

### Ecological applications of *VEBA* workflows

**Fig. 5:**
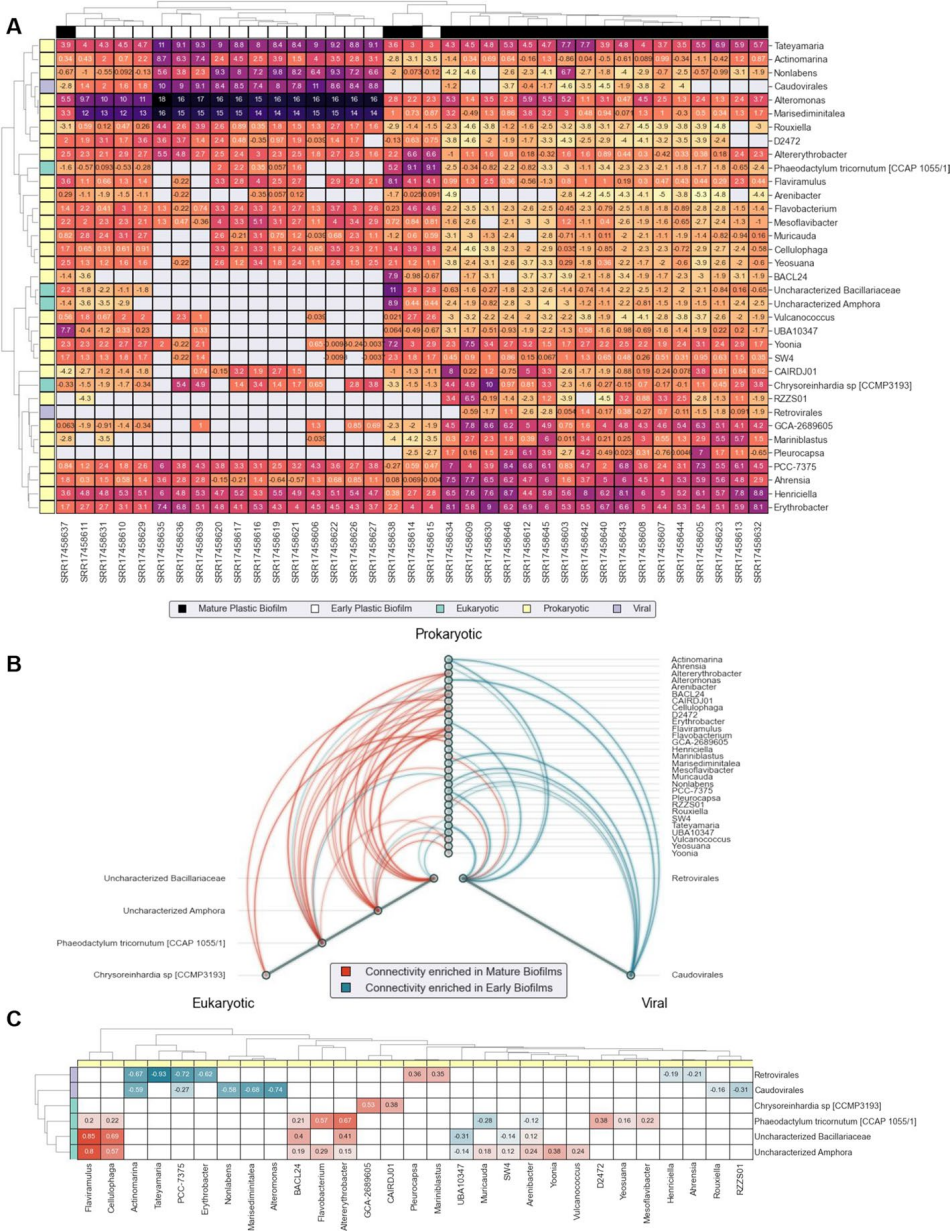
Compositional data analysis of Plastisphere. **A** Clustered abundance heatmap of CLR values using Aitchison distance and 1 − *ρ* as sample and taxon distance metrics, respectively, followed by average linkage hierarchical clustering. **B** Differential co-occurrence hive network between mature and early plastic biofilms using *ρ* proportionality as the association matrix with positive and negative differential connectivity colored as red and blue, respectively. **C** Heatmap of differential connectivity values in the hive network

One of the biggest advantages of coassembly-based metagenomics over sample-specific approaches is that the resulting contigs, and by extension genes and MAGs, are directly comparable across all samples used to generate the coassembly while the latter produces disjoint contigs that are specific to each sample. *VEBA* uses the strengths of sample-specific and coassembly approaches by clustering and aggregating genomic features providing an avenue for comparing features across samples; a necessity in downstream analytical methods. To demonstrate the ecological applications of *VEBA*’s multi-domain binning, clustering, and feature compression approaches, we implemented a clustered abundance heatmap (Fig. 5A) and compositionally-valid differential co-occurrence network to investigate differential connectivity in mature and early plastic biofilms (Fig. 5B, C).

In the clustered abundance heatmap, the most obvious trend is that samples naturally group by either mature or early plastic biofilms. Another defining characteristic is that the mature plastic biofilm samples have greater taxonomic richness and are not dominated by any one taxa as is the case in early biofilm samples which are dominated by *Caudovirales* viruses and *Alteromonas*, *Marisedimintalea*, *Nolabens*, and *Tateymaria* bacteria. In particular, *Alteromonadaceae* genera (e.g., *Alteromonas* and *Marisediminitalea*) are the most abundant organisms in the early plastic biofilm community which agrees with read-based analyses of the original study [102] and previous research [126]. Many of the early plastic biofilm samples completely lack diatoms, pelagophytes, and retroviruses that are both abundant and prevalent in mature biofilms. Another characteristic of the mature plastic biofilm grouping is that almost every sample contains *Retrovirales* and only a few contain *Caudovirales* (though, at low abundance) suggesting these may influence community dynamics.

The most notable trend for the differential co-occurrence network is that prokaryotes overall have stronger co-occurrence with viruses in early plastic biofilms and transition to co-occurring more strongly with eukaryotes in mature biofilms (Fig. 5B, C). The only prokaryotes that have an increased co-occurrence in mature biofilms with any virus in the network are *Mariniblastus* and *Pleurocapsa;* an enriched connectivity to *Retrovirales.* While no RNA viruses are known to infect *Mariniblastus* or *Pleurocapsa,* RNA viruses have been well documented in eukaryotic phytoplankton [127] for which these bacteria co-occur. *Mariniblastus* have been isolated from the surface of algae [128] and associations between cyanobacteria and diatoms have been well characterized [129] suggesting an indirect association rather than a host/virus relationship.

*Alteromonadaceae* genera co-occur strongly with *Caudovirales* phages*.* Phage infection may give rise to genetic diversity amongst *Alteromonadaceae* and their ability to colonize early plastic biofilms which may be the case in plastic biofilms with 12 *Alteromonas macleodii* MAGs*, 9 Marisediminitalea aggregate* MAGs, and 2 uncharacterized species of *Alteromonas* recovered. As mentioned in the original study [102], *Alteromonas* and *Marisediminitalea* have large flexible genomes and contain a broad metabolism suited to colonize diverse substrates and metabolize various carbon sources [130, 131].

*Flaviramulus* and *Cellulophaga* co-occur strongly with diatoms in the mature plastic biofilms. Diatoms and *Flaviramulus* have long been known to establish marine biofilms on artificial surfaces [132, 133]. Further, *Cellulophaga* strains with potent extracellular enzymic activity have been isolated from the surfaces of the chain-forming sea-ice diatoms [134] suggesting a similar mechanism with the *Cellulophaga tyrosinoxydans* species associating with diatoms in mature plastic biofilms. However, exploring these relationships is speculative and not the primary directive of this demonstration of applications.

## Conclusions

In this study, we provide a software suite that allows for the in silico recovery of microorganisms from all domains of life by integrating cutting edge algorithms in novel ways. *VEBA* fully integrates both end-to-end and task-specific metagenomic analysis in a modular architecture that minimizes dependencies and maximizes productivity. *VEBA’s* unsupervised clustering at the genomic and protein level provides a means to have the best of both worlds in terms of sample-specific and coassembly-based assembly-centric metagenomics; that is, biologically-relevant (i.e., less composite) genomes are recovered while also being comparable across samples. This clustering also provides a means to use feature engineering to aggregate counts from groups of related features to reduce dimensionality for downstream analysis. In addition, *VEBA* outputs machine-readable identifier mapping tables that can be used for accounting of features along the biological feature hierarchy (contig—MAG—SLC and ORF—SSO).

Using sample-specific binning followed by pseudo-coassembly binning of concatenated unbinned contigs from multiple samples was demonstrated here to recover far more quality MAGs than non-iterative modes. *VEBA* does not introduce a novel binning algorithm but instead builds upon established workflows and reuses discarded contigs in novel ways. For instance, *VEBA* utilizes *DAS Tool* for consensus binning of *MaxBin2*, *MetaBAT2*, and *CONCOCT* as the base for recovering prokaryotic genomes; a workflow that has been well established and rigorously benchmarked by previous research [29, 34]. The merit of iterative binning is apparent when considering that additional genomes are recovered in all 3 case study datasets that would have been discarded using non-iterative methods. This merit is also apparent with pseudo-coassembly binning where we recovered additional high-quality genomes, but we recognize that with any coassembly-based methods the possibility of recovering composite genomes increases. To account for this property, we add pseudo-coassembly binning solely as an optional feature that can be implemented for users that have datasets with highly similar biological samples believed to contain overlapping microbial communities. Regardless, the same strict quality assessment via *CheckM* is performed for both sample-specific and pseudo-coassembly approaches.

*VEBA* was designed to be modified and updated as new peer-reviewed software becomes available. For instance, the standardized output of the prokaryotic binning procedure could allow for additional binning algorithms to be added or swapped out. There are several adaptations planned for future releases of *VEBA* once new software has been peer-reviewed or existing software has been updated. The first adjustment would be to update *CheckM* to *CheckM2* [135] which is currently in preprint phase. Although *CheckM* version 1 can handle CPR, it cannot do so with the recommended *lineage_wf* directly but instead with a separate manual workflow. *VEBA’s* prokaryotic binning module automates the *lineage_wf*, *GTDB-Tk* classification, the manual *CheckM* CPR workflow, and concatenates the output so users can have a seamless experience without manually rerunning algorithms, subsetting tables, and updating quality assessments (see *Methods*). *CheckM2* is expected to handle this directly and will be implemented in *VEBA* once peer-reviewed and available via *Bioconda*. Another potential modification will be the incorporation of *EukRep* in addition to *Tiara* for eukaryotic classification. The decision to use *Tiara* over *EukRep* in the initial release was based the following considerations: (1) *Tiara* is reported to outperform *EukRep* in terms of prediction accuracy and calculation time [61, 136]; (2) *Tiara* has an option to output prediction probability vectors (*EukRep* does not) allowing probabilities to be aggregated for bin-level predictions; (3) *Tiara* is designed to handle eukaryotic organelles; and (4) the current *EukRep v0.6.7* version backend models are dependent on a deprecated *Scikit-Learn* version 0.19.2 (https://github.com/patrickwest/EukRep/issues/14) forcing users to downgrade their environment. If future *EukRep* versions can address these issues, *VEBA* will certainly add it as an additional option for users*.* Lastly, there are two software packages under active development designed specifically for eukaryotic metagenomics that are also in preprint phase. The first software package is *EukMetaSanity* [137] which is a structural and functional annotation algorithm for eukaryotic MAGs. While *EukMetaSanity* is expected to produce more robust gene modeling than *MetaEuk*, the dependency of restrictive licensing software (e.g., *GeneMark* and *RepeatMasker*) conflicts with the objectives of *VEBA* in avoiding the use of limited restriction software. The second software package is *EukHeist* [138] which performs similar operations to *VEBA’s* eukaryotic binning module but uses *EukRep* at the contig level instead of the MAG level and couples binning with assembly. Once peer-reviewed, future versions of *VEBA* can incorporate workflows built around the input and output of *EukMetaSanity* and *EukHeist* that can synergize the benefits of *VEBA* and external software packages. However, eukaryotic genomes binned with *EukHeist* and/or genes modeled with *EukMetaSanity* are already supported by *VEBA*’s mapping-based modules (*coverage.py, mapping.py, index.py*), phylogenetic inference module (*phylogeny.py*), genomic/orthogroup clustering module (*cluster.py*), and protein-product annotation module (*annotate.py*); this accessibility holds true for any custom genomes or gene models either binned or downloaded from some repository.

Despite the utility of *VEBA* and the backend software, there are several limiting factors that must be addressed by future research. One limiting factor in genome-resolved microeukaryotic metagenomics is the lack of consensus binning tools that can handle microeukaryotic lineages. However*, DAS Tool* [34] is currently working on implementing custom marker sets which may be available in future versions (https://github.com/cmks/DAS_Tool/issues/69). Ideally, this type of workflow would be combined with *BUSCO’s* lineage-specific marker sets to handle lineage-specific completeness and contamination quality assessment. Another limiting factor for both microeukaryotic and viral metagenomics is the lack of taxonomy classification with the same rigor as *GTDB-Tk*. Currently, the only peer-reviewed tool designed for eukaryotic taxonomy classification is *EUKulele* [139] but there were several barriers we experienced when attempting to incorporate *EUKulele*. First, many of the existing *EUKulele* databases are targeted towards marine ecosystems, thus, not practical for alternative environments (e.g., human microbiomes, soil, built-environments), contain eukaryotes which would not be expected to be binned in a metagenome, and contain prokaryotic genomes that increase computational resource demand. Second, when trying to build a custom *EUKulele* database using *VEBA’s* microeukaryotic protein database as a reference, we experienced fatal errors that could not be directly diagnosed but were likely due to the dependency of supergroup and division fields that were missing for certain taxa. If we are able to resolve these issues in collaboration with *EUKulele* developers, then *VEBA* can incorporate an option to leverage *EUKulele* as an alternative to *VEBA’s* default eukaryotic classification module.

To fully understand an ecosystem and how changes within an ecosystem are associated with sustainability or human health, we must consider all members of the microbiome including eukaryotes and viruses in addition to the already established precedence of prokaryotes. As of April 2022, there are 1,250 protist genome assemblies publicly accessible through NCBI and only 23 of these genomes are considered complete. Although there has been an emerging interest in microeukaryotic metagenomics, there has not been a full awakening because the type of industry-standard workflow and convenience that exists for prokaryotic metagenomics has not been available for microeukaryotic metagenomics. Opportunely, the advent of *MetaEuk* for gene modeling and the recent updates to *BUSCO* for lineage-specific genome quality assessment used in parallel with domain-agnostic binning algorithms (*MetaBAT2*, *CONCOCT*) has made the quest for microeukaryotic metagenomics more accessible to the modern bioinformatician which are implemented in the eukaryotic workflow of *VEBA*. While short-read technologies may not yield complete genomes due to repeat region resolution, non-coding complexity, and multiple chromosomes, they certainly link taxonomy with function which is critical for characterizing ecological changes related to climate change and human disease. Further, these draft genomes sourced from metagenomes may serve as references for hybrid short/long-read technologies to polish and complete genomes for organisms that cannot be cultured.

The recovery of phages in communities dominated by a particular genus such as *Staphylococcus* in the *Netherton* microbiome and *Alteromonas* in the *Plastisphere* microbiome could have novel applications for synthetic biology and bioengineering. In the case of the Netherton syndrome, an untreatable disease, these phages can be assessed for host specificity and their potential to target specific strains of *Staphylococcus* that contribute to diseased phenotypes. Recent research suggests that phage therapy could be used in the fight against antimicrobial resistance [140] and skin disorders such as psoriasis [141].

In the context of the human microbiome, prior research has provided vast insight into which prokaryotes are considered commensals, mutualists, or parasites. While the ecology of some pathogenic microeukaryotes is well characterized, this is not the case for commensal and mutualistic microeukaryotes. This *modus operandi* is reminiscent of bacteriology before early microbiome studies where most bacteria associated with humans were considered to be harmful [142]. Thus, our understanding of microeukaryotic roles in ecological communities contains a blind spot from the bias of funded research towards pathogenic organisms; understandably given their direct relation to disease. In the context of biotechnology, this gap in our knowledge base may contain organisms and mechanisms relevant for biomedical applications or sustainability.

The current culture of biological science and research funding has been hyper focused on acquiring new biological samples for solving existing problems. While sequencing new biological material is essential in progressing science, this paradigm tends to overlook the undiscovered wealth available in existing datasets that can be economically reevaluated using modern methodologies such as *VEBA*. We demonstrated that our method can be applied to effectively mine out new information and uncharacterized organisms from existing published datasets. Large-scale efforts to sequence the entirety of life is not trivial by any means. As stated eloquently by Lewin and colleagues, “while recognizing that it may not be feasible to obtain samples for every species, pragmatism does not negate the primary scientific and societal need for trying to do so” [6]. The time has come to maximize the amount of information acquired from new and existing biological datasets by using iterative methodologies and extending the precedent of prokaryotes to eukaryotic organisms and viruses.

## Supplementary Information

**Additional file 1. Supplemental Table 1**. Contains NCBI SRA identifiers, BioProject identifiers, the number of paired reads, and the number of prokaryotic, eukaryotic, and viral MAGs.

**Additional file 2. Supplemental Table 2**. Contains taxonomy source identifier mappings to taxonomic class, order, family, genus, and species for *VEBA*'s microeukaryotic protein database.

**Additional file 3. Supplemental Table 3**. Contains dataset, domain, assembly statistics, taxonomy, and genome quality for 948 MAGs.

## Data Availability

The case study datasets analyzed during the current study are available in NCBI’s SRA repository under the following BioProject identifiers: PRJNA777294, PRJEB20421, and PRJNA551026. *VEBA* modules, algorithms, and utility scripts are open-sourced on GitHub (https://github.com/jolespin/veba). *VEBA* microeukaryotic protein database (10.6084/m9.figshare.19668855.v1), profile HMM marker database (10.6084/m9.figshare.19616016.v1), and case studies (10.6084/m9.figshare.20263974.v1) are available on FigShare. Case study data includes MAG assemblies, gene models, gene annotations, taxonomy classifications, clusters, and counts tables for each dataset.

## Abbreviations

ANI: Average nucleotide identity
CLR: Center log-ratio
CPR: Candidate phyla radiation
EBP: Earth BioGenome Project
EMP: Earth Microbiome Project
FCR: Feature compression ratio
HMP: Human Microbiome Project
GOS: Global Ocean Sampling
MAG: Metagenome-assembled genome
ORF: Open reading frame
SLC: Species-level cluster
SSO: SLC-specific orthogroup
VEBA: Viral Eukaryotic Bacterial Archaeal
